# Metagenomic Sequencing of Wastewater from a South African Research Farm

**DOI:** 10.1128/MRA.01323-18

**Published:** 2018-10-25

**Authors:** Briallen Lobb, Anthony A. Adegoke, Kesen Ma, Andrew C. Doxey, Olayinka A. Aiyegoro

**Affiliations:** a Department of Biology, University of Waterloo, Waterloo, Ontario, Canada; b Department of Microbiology, Faculty of Science, University of Uyo, Uyo, Akwa Ibom State, Nigeria; c GI Microbiology and Biotechnology Section, Agricultural Research Council–Animal Production, Irene, South Africa; Broad Institute of MIT and Harvard

## Abstract

We sequenced wastewater effluent from the Agricultural Research Council–Animal Production in South Africa that conducts studies on livestock health and farm ecology. Thauera, Oscillibacter, and Pseudomonas were the most abundant genera within the community. Thirty-one different antibiotic resistance genes were identified, 10 of which are associated with tetracycline resistance.

## Abstract (precis)

We sequenced wastewater effluent from the Agricultural Research Council–Animal Production in South Africa that conducts studies on livestock health and farm ecology. Thauera, Oscillibacter, and Pseudomonas were the most abundant genera within the community.

## ANNOUNCEMENT

Antibiotics are used to promote growth and manage disease in livestock at Agricultural Research Council–Animal Production in South Africa. However, the spread of antibiotic resistance is a pervasive concern. Waste from farm animals has been shown to spread antibiotic-resistant bacteria, sometimes due to selective pressure found in antibiotic-dosed livestock (1–3). One of a farm’s effluents, wastewater, is a documented reservoir of antibiotic resistance genes that could transfer to human pathogens (4–6). Wastewater is also known to contain animal pathogens, some of which are opportunistic and can spread zoonoses (7, 8). Sequencing of the wastewater microbiome can help identify pathogenic species that might exist on the institute’s farm and detect antibiotic resistance genes that may be active in these microbial communities.

The metagenome was created from expended water taken from Agricultural Research Council–Animal Production (ARC-AP) in Irene, South Africa. A 1-liter composite sample was created by combining five 200-ml samples collected from different wastewater gutters in the pig facility. The composite sample was centrifuged at 3,500 rpm for 10 min at room temperature to separate the biomass and water. The water was filtered to trap microbes, and DNA was extracted from the pellet on the filter paper. The DNA extraction was done using the FastDNA Spin kit for water (MP Biomedicals, Solon, OH, USA) and the FastPrep apparatus, according to the instructions given by the manufacturer. The DNA was sequenced with the Illumina HiSeq platform and the Illumina HiSeq reagent v3. A total of 28,540,348 read pairs with an average read length of 119 bp each were generated. The reads were trimmed with Sickle version 1.33 (9) and Trim Galore! version 0.5.0 (10) and then assembled using MEGAHIT version 1.1.2 (11), resulting in 58,129 contigs longer than 1 kb. The total assembly length was 311,492,658 bp, with an *N*_50_ value of 861 bp. Prodigal version 2.6.3 with the -p meta option (12) was used next, facilitating the prediction of 612,922 coding sequences.

A profile of the community based on taxonomic marker genes was constructed with MetAnnotate using the usearch option (13). An abundance for each marker gene hit was calculated using Bowtie 2 version 2.3.4.2 (14), SAMtools version 1.9 (15), and BEDtools version 2.27.1 (16). The abundance of each gene was calculated as the average coverage per base pair across the coding sequence. The most common genera present (based on the average abundance across all taxonomic markers) are Thauera (19%), Oscillibacter (7%), Pseudomonas (6%), Prevotella (5%), and Bacteroides (3%).

A blastp search of the homolog models in the Comprehensive Antibiotic Resistance Database (CARD) (17) identified 31 different antibiotic resistance genes that passed CARD’s strict score threshold. Ten of these genes are predicted to confer tetracycline resistance, and 5 genes are predicted to confer streptomycin resistance. Annotation with the Kyoto Encyclopedia of Genes and Genomes (KEGG) database (18) using GhostKOALA revealed complete pathways for tetracycline, streptomycin, aminoglycoside, cationic antimicrobial peptide (CAMP), vancomycin, and macrolide resistance, with near-complete pathways for beta-lactam, erythromycin, fluoroquinolone, and lincosamide resistance.

### Data availability.

This whole-genome shotgun project has been deposited at GenBank under the accession number QXGG00000000. The version described in this paper is version QXGG01000000. The raw reads were deposited in the Sequence Read Archive (SRA) under the accession number SRP159184.
